# Bacterial communities and metabolic activity of faecal cultures from equol producer and non-producer menopausal women under treatment with soy isoflavones

**DOI:** 10.1186/s12866-017-1001-y

**Published:** 2017-04-17

**Authors:** Lucía Guadamuro, Anja B. Dohrmann, Christoph C. Tebbe, Baltasar Mayo, Susana Delgado

**Affiliations:** 1 Department of Microbiology and Biochemistry, Instituto de Productos Lácteos de Asturias (IPLA), Consejo Superior de Investigaciones Científicas (CSIC), Asturias, Spain; 2 Thünen-Institut für Biodiversität, Bundesforschungsinstitut für Ländliche Räume, Wald und Fischerei, Braunschweig, Germany

**Keywords:** Faecal slurry cultures, Faecal fermentation, Isoflavone metabolism, Equol, Intestinal microbiota, High-throughput sequencing

## Abstract

**Background:**

Isoflavones are polyphenols with estrogenic activity found mainly in soy and soy-derived products that need to be metabolised in the intestine by the gut bacteria to be fully active. There is little knowledge about isoflavone bioconversion and equol production in the human intestine. In this work, we developed an in vitro anaerobic culture model based on faecal slurries to assess the impact of isoflavone supplementation on the overall intestinal bacterial composition changes and associated metabolic transformations.

**Results:**

In the faecal anaerobic batch cultures of this study bioconversion of isoflavones into equol was possible, suggesting the presence of viable equol-producing bacterial taxa within the faeces of menopausal women with an equol producer phenotype. The application of high-throughput DNA sequencing of 16S rRNA gene amplicons revealed the composition of the faecal cultures to be modified by the addition of isoflavones, with enrichment of some bacterial gut members associated with the metabolism of phenolics and/or equol production, such as *Collinsella*, *Faecalibacterium* and members of the *Clostridium* clusters IV and XIVa. In addition, the concentration of short-chain fatty acids (SCFAs) detected in the isoflavone-containing faecal cultures was higher in those inoculated with faecal slurries from equol-producing women.

**Conclusions:**

This study constitutes the first step in the development of a faecal culturing system with isoflavones that would further allow the selection and isolation of intestinal bacterial types able to metabolize these compounds and produce equol in vitro. Although limited by the low number of faecal cultures analysed and the inter-individual bacterial diversity, the in vitro results obtained in this work tend to indicate that soy isoflavones might provide an alternative energy source for the increase of equol-producing taxa and enhancement of SCFAs production. SCFAs and equol are both considered pivotal bacterial metabolites in the triggering of intestinal health-related beneficial effects.

**Electronic supplementary material:**

The online version of this article (doi:10.1186/s12866-017-1001-y) contains supplementary material, which is available to authorized users.

## Background

Isoflavones are polyphenols with estrogenic activity found at relatively high concentration in soy and soy-derived products. Epidemiological studies suggest high intakes of isoflavones to be associated with fewer and less intense menopause symptoms, and a reduced incidence of hormone-dependent and aging-associated diseases such as osteoporosis, cardiovascular diseases and cancer [1]. Although there is a growing body of scientific evidence showing beneficial effects in counteracting symptoms such as hot flushes and vasomotor reactions in menopausal women [2], the European Food Safety Authority (EFSA) has recently refuted health claims about the role of isoflavones in body functions [3]. This may be so because the functionality of isoflavones depends on their bioavailability and their conversion into (more) active metabolites within the intestinal tract. In nature, isoflavones are mostly (>80%) conjugated with sugars as isoflavone-glycosides (daidzin, genistin, glycitin) with low availability and bioactivity [4]. For full activity, aglycones (daidzein, genistein, glycitein) need to be released from their glycosides and, occasionally, metabolized to more potent metabolites, such as equol (produced from daidzein). Equol is the isoflavone-derived compound with the strongest estrogenic activity and antioxidant capacity, mechanisms by which isoflavones seem to provide the purported beneficial health effects [5]. The conversion of isoflavones into equol appears to be mainly carried out by intestinal microorganisms, especially bacteria inhabiting the distal portion of the gut [6]. Both, traditional and molecular methods have revealed marked individual diversity in intestinal bacterial communities. Such differences in the bacterial community composition may, in fact, influence the fate of isoflavone metabolic transformations [7, 8], which could ultimately contribute to differences in the physiological response to isoflavone treatment.

In that sense, only 30–50% of Western individuals produce equol [5], and only these might fully benefit from the positive health effects of isoflavone consumption. The metabolism of isoflavones involves several steps mediated by enzymes provided by different bacterial taxa [7, 8]. Even though scientific information is accumulating on the microorganisms producing equol, their enzymes and the metabolic routes involved, our current knowledge is still limited [6, 9, 10]. Most of the equol-producing bacteria characterized so far belong to the family *Coriobacteriaceae* [7]. However, it is not yet clear whether this family is the only intestinal group acting on isoflavones and producing equol.

Previously, the metabolism of daidzein by faecal bacterial consortia has been microbiologically characterized by conventional culturing methods [11–13]. The availability of high-throughput DNA sequencing techniques opens new potentials to tracking changes in the bacterial communities during isoflavone supplementation in both in vitro and in vivo systems. A better knowledge about the identity and individual variability of the intestinal bacteria that metabolize isoflavones and convert them into equol, would provide an important step for developing strategies to increase bioavailability and concentration of active compounds, e.g. by supplying suitable equol-producing probiotic bacteria.

In this study, anaerobic batch cultures of faecal samples from equol producer and non-producer menopausal women (as determined by urine equol excretion of >1000 nM) under treatment with soy isoflavones were performed, using media with and without isoflavones, to examine which bacteria take benefit from soy isoflavones and could be involved in equol production. PCR- denaturing gradient gel electrophoresis (DGGE) and high-throughput DNA sequencing were used to determine whether faecal cultures grown in the presence of isoflavones showed any change in their bacterial community composition and/or structure. These molecular analyses, based on PCR-amplified partial 16S rRNA gene sequences, were complemented by metabolic profiling of the culture supernatants using ultra-high-performance liquid chromatography (UHPLC) and gas chromatography (GC). This approach allowed the suggestion of links between structural responses in bacterial community compositions to metabolic activities.

## Methods

### Stool samples from isoflavone-treated menopausal women

Menopausal women who had been receiving treatment for 6 months with 80 mg/day of an isoflavone concentrate (Fisiogen; Zambon, Bresso, Italy) were recruited. Stool samples were obtained from four women whose faecal microbiota had been characterized and whose equol producer status during isoflavone treatment had been determined in a previous study [14]. Three of the women (WC, WG and WP) were equol producers (urine equol >1000 nM as defined by Rowland et al. [15]), while the fourth (WE) was a non-producer (<10 nM in urine). Faeces were collected and transported to the laboratory as previously described [14].

### Faecal batch cultures

Ten-fold faecal dilutions were prepared by homogenizing 1 g of faeces in 9 ml of a pre-reduced phosphate buffer saline solution (PBS) under strict anaerobic conditions (80% N_2_, 10% CO_2_, 10% H_2_) in a Whitley DG500 Workstation anaerobic chamber. A 10% (*v*/*v*) aliquot of the resulting faecal slurry was used to inoculate the medium for colonic bacteria (MCB) described by van der Meulen et al. [16], modified to have a lower glucose content (2 g/l) (hereafter referred to as mMCB). This medium was prepared without and with a concentration of 160 mg/l commercial isoflavone supplement (Fisiogen) by dissolving two capsules per liter of medium. One capsule of Fisiogen contains, as stated in the package, 80 mg of isoflavone of which 55–72% is genistin/genistein and 28–45% other soy isoflavones. It also includes as excipients E341, E460, E468, E551, E472, E464, E904, E171, and E124. The isoflavone-containing medium is hereafter referred to as mMCB_ISO_. These faecal primary cultures thus prepared were incubated at 37 °C in fermentation flasks with mild stirring (250 rpm) for 24 h under anaerobic conditions. To find out whether production of equol in vitro was maintained in successive subculturings, primary cultures in mMCB_ISO_ were used as inocula (10% *v*/*v*) for a second round of cultivation in fresh mMCB_ISO_. These new cultures were incubated as above and referred to as secondary cultures.

Positive controls for isoflavone conversion and equol production were obtained by inoculating (at 10%) mMCB_ISO_ with the strains *Slackia isoflavoniconvertens* DSM 22006 or *Slackia equolifaciens* DSM 24851. Both these strains were grown under anoxic conditions in Gifu Anaerobic Medium (GAM, Nissui Pharmaceutical, Tokyo, Japan) supplemented with 0.5% arginine (Merck, Darmstadt, Germany). After overnight culturing, cell of the two strains were washed twice in pre-reduced PBS before inoculating the media (mMCB and mMCB_ISO_) or the faecal cultures and incubated as above.

### Detection and quantification of equol and its isoflavone precursors

Equol and its isoflavone precursors [daidzin (daidzein-7-O-glucoside) and daidzein] were measured using a UHPLC procedure based on a method for equol determination in urine [17]. Briefly, after 24 h incubation, faecal cultures were centrifuged at 800 rpm for 10 min, duplicate samples of 3 ml of each supernatant collected, and their isoflavone content extracted using Bond Elute-C18 solid-phase cartridges (Agilent Technologies, Santa Clara, CA, USA). After drying, organic extracts were dissolved in 100 μl of HPLC grade methanol, and 1 μl of each sample injected into the UHPLC apparatus (Waters, Palo Alto, CA, USA). Samples were analysed in duplicate. Equol was quantified using a fluorescence detector (excitation 280 nm, emission 310 nm), while daidzin and daidzein were identified with a photodiode array (PDA) detector by comparison with their retention times and spectral characteristics at 260 nm. Quantification was performed against calibration curves prepared using commercial standards (all from Sigma-Aldrich, St. Louis, MO, USA).

### Short chain fatty acids determination

Short chain fatty acids (SCFAs) in the primary and secondary cultures were determined by GC. Culture supernatants were centrifuged for 10 min at 800 rpm, and then for 10 min further at 13,200 rpm, before filtering through 0.45 μm PTFE filters. The filtered samples were then mixed 10:1 (*v*/*v*) with 1 mg/ml of 2-ethyl butyric acid (Sigma-Aldrich) dissolved in methanol as an internal standard. A chromatographic system composed of 6890 N GC apparatus (Agilent Technologies) connected to a flame ionization detector was used to identify and quantify the SCFAs as described elsewhere [18]. All samples were analysed in triplicate. The Shapiro–Wilk test was initially used to check for the normal distribution of the data. Since the data were not normally distributed, we used the non-parametric Wilcoxon signed-rank test to examine the differences in the content of SCFAs between the faecal cultures with (mMCB_ISO_) and without isoflavones (mMCB). This statistic test is used to assess differences between related groups of samples and in our case the same faecal cultures were analysed under two different conditions (mMCB versus mMCB_ISO_). All these calculations were performed using the SPSS software v.22.0.

### Microbial community analysis

#### DNA extraction

Total bacterial DNA was extracted from the faecal primary and secondary cultures using the phenol-chloroform-based protocol of Zoetendal et al. [19] with modifications as described elsewhere [20]. The main modification consisted in the addition of an enzymatic lysis step before mechanical disruption in a FastPrep FP120 apparatus (Qbiogene, Carlsbad, CA, USA). After precipitation and drying, DNA samples were suspended in 100 μl of sterile molecular biology grade water (Sigma-Aldrich).

#### PCR-denaturing gradient gel electrophoresis (DGGE) analysis

Total DNA from the faecal cultures with and without isoflavones was used as a template for amplification of the variable region V3 of the 16S rRNA gene by PCR using the universal prokaryotic primers F357-GC and R518 [21]. DGGE electrophoresis was performed as described elsewhere [14]. DNA from *S. equolifaciens* and *S. isoflavoniconvertens* was used to provide DGGE markers. Genomic DNA from these strains was obtained from overnight cultures in GAM + 0.5% arginine using the GenElute Bacterial Genomic DNA kit (Sigma-Aldrich) and subsequently employed in PCR amplifications. Amplicons were purified using GenElute PCR Clean-Up columns (Sigma-Aldrich) and mixed in equal amounts to obtain a DGGE marker.

#### High-throughput DNA sequencing and analysis of 16S rRNA gene amplicons

DNA samples obtained from the primary and secondary cultures were subjected to PCR amplification of the variable region V4-V5 of the 16S rRNA gene using the universal prokaryotic primers 515F and 909R [22]; these were designed to include Illumina adapters. Double indexed amplicons were generated by the protocol of Caporaso et al. [23] with minor modifications. In brief, DNA from the samples was extracted in duplicate and amplified in triplicate; in total, 72 amplification reactions were obtained. These were subsequently paired-end sequenced in an Illumina Miseq System (Illumina, San Diego, CA, USA) by the StarSeq Company (Mainz, Germany), and treated as independent replicates.

Bioinformatic analysis was performed using the Mothur software package (v.1.34.1) following the MiSeq Standard Operating Procedure (SOP) [24]. Briefly, sequences longer than 380 bp in length, or shorter than 370 bp, and those containing ambiguous base pairs or homopolymers regions of >8 bp were removed. All other sequences were aligned using the SINA alignment service of the SILVA 16S rRNA sequence database. Chimera removal was performed using the UCHIME algorithm [25]. A random subset of 35,000 sequences per sample was used to balance numbers of reads among samples. Sequences were then clustered into operational taxonomic units (OTUs) using a 0.03 dissimilarity cut-off. Sequences were taxonomically classified using the Ribosomal Database Project (RDP) database. The Bayesian classifier with an 80% confidence threshold was used in the taxonomic assignment with the genus level as the lowest taxonomic unit considered. The MOTHUR program was also used to perform weighted UniFrac analysis, which was employed to assess the similarity of the microbial communities between samples. Construction of a heatmap was performed using the R statistical software. Clustering was accomplished using the complete linkage method with Euclidean distance measure. Multivariable statistical analysis was performed by principal coordinates analysis (PCoA) and non-metric multidimensional scaling (NMDS) with two dimensions. Differences in the microbial composition of faecal cultures with and without isoflavones were sought by analysis of molecular variance (AMOVA) and analysis of similarities (ANOSIM). The identification of differentially abundant taxa was assessed using the Metastats software [26]. Multiple hypothesis tests were adjusted using the false discovery rate (FDR) correction [27]; an FDR *q*-value threshold of 0.25 was used to identify significant differences. In an attempt to assign the differential OTUs at the species level, manual sequence comparisons were performed against the Greengenes 16S rRNA gene database.

## Results

### Isoflavone conversion and equol production

The uninoculated medium with isoflavones, mMCB_ISO,_ was shown by UHPLC to contain three times more conjugated precursor daidzin that its corresponding aglycone daidzein (Table 1). As expected, equol was never detected in the absence of isoflavones in the cultures. In the medium inoculated with *S. equolifaciens* DSM 24851, used as a positive control for equol production, daidzin levels were maintained while daidzein was transformed and converted to equol, reaching 2 μg/ml after 24 h incubation. In the primary faecal cultures from the three equol producers (WC, WG and WP) comparable amounts of equol to that produced by the positive control were recorded (1.40–3.07 μg/ml). Congruently, daidzin levels were drastically reduced in these cultures, indicating glycosidase activity from faecal bacteria towards this isoflavone glycoside. In the primary culture from the non-producing woman (WE), daidzin was also shown to decrease while daidzein accumulated, but no equol was found (limit of quantification 0.002 μg/ml). As expected, in the secondary culture from this woman equol was not detected either. In contrast, equol production was maintained to some extent in the secondary cultures from two of the three equol producers (WG and WP).

**Table 1:** Isoflavone levels (μg/ml) in faecal cultures from equol-producing and non-producing women in mMCB_ISO_ medium

Sample	Isoflavone	Primary cultures	Secondary cultures
WC^a^	Daidzin	0.24 ± 0.09	24.69 ± 5.10
	Daidzein	1.74 ± 0.07	under LOQ^c^
	Equol	2.62 ± 0.04	under LOQ
WG^a^	Daidzin	0.05 ± 0.02	0.12 ± 0.06
	Daidzein	under LOQ	under LOQ
	Equol	3.07 ± 0.24	7.67 ± 0.64
WP^a^	Daidzin	0.09 ± 0.04	0.18 ± 0.07
	Daidzein	0.02 ± 0.01	under LOQ
	Equol	1.40 ± 0.06	4.02 ± 0.35
WE^b^	Daidzin	under LOQ	under LOQ
	Daidzein	24.10 ± 3.32	45.57 ± 5.46
	Equol	under LOQ	under LOQ
Uninoculated culture media	Daidzin	16.83 ± 2.89	N.A.
	Daidzein	5.57 ± 1.19	N.A.
	Equol	under LOQ	N.A.
*Slackia equolifaciens*	Daidzin	16.53 ± 3.68	N.A.
	Daidzein	0.06 ± 0.05	N.A.
	Equol	2.01 ± 0.10	N.A.

*N*.*A.* not applicable, *mMCB*_*ISO*_ modified medium for colonic bacteria supplemented with isoflavones

^a^equol-producing woman

^b^equol non-producing woman

^c^ *LOQ* Limit of quantification (0.002 μg/ml for equol; 0.004 μg/ml for daidzein and 0.014 for daidzin)

### SCFA production

**Fig. 1:**
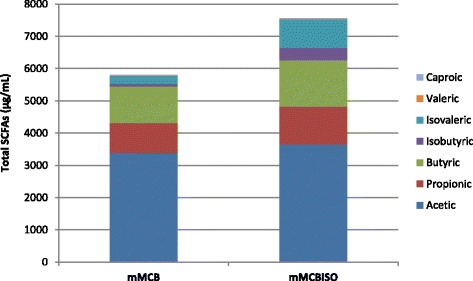
Production of short chain fatty acids (SCFAs). Comparison of total SCFAs production by the primary faecal cultures in media without (mMCB) and with (mMCB_ISO_) isoflavones

Comparing the results obtained in the mMCB medium, total SCFA production was greater in the faecal cultures containing isoflavones (mMCB_ISO_) (Fig. 1). The concentrations of the major SCFAs (acetic, propionic and butyric acids) were particularly enhanced in the primary faecal cultures whose inocula came from the equol-producing women (Table 2); significant increases were seen for propionic acid and isobutyric and isovaleric acids (the latter two are minority, branched chain fatty acids [BCFAs]) (*p* < 0.05). In contrast, in the cultures from the equol non-producing woman (WE), no differences between the corresponding mMCB_ISO_ and mMCB were seen for major or minor SCFAs in the primary cultures. Consequently, the acetic/propionic ratio for this faecal culture was five times higher than that recorded for the equol producers; indeed, the concentration of propionic and butyric acids for this culture were much lower than those recorded in the primary cultures of the equol-producing women. SCFAs production in the secondary cultures from these women was maintained to some extent, but with a major reduction in the concentration of isovaleric acid.

**Table 2:** Short chain fatty acid (SCFA) production (mean ± standard deviation) in faecal cultures

Equol status	Sample		SCFA (μg/ml)							
			Acetic	Propionic	Butyric	Acetic/Propionic	Isobutyric	Isovaleric	Valeric	Caproic
Producers (*n* = 3)	Primary cultures	mMCB	3630.78 ± 364.76	1204.29 ± 392.01	1420.04 ± 749.28	3.25 ± 0.88	97.18 ± 81.83	312.51 ± 208.9	24.52 ± 18.66	5.71 ± 5.29
		mMCB_ISO_	4063.17 ± 322.93	1559.54 ± 325.14*	1859.97 ± 570.4	2.74 ± 0.76*	471.72 ± 5.28*	1114.00 ± 17.79*	27.11 ± 7.9	3.82 ± 1.37
	Secondary cultures	mMCB_ISO_	3334.11 ± 655.9	1545.72 ± 632.46	1649.15 ± 189	2.34 ± 0.53	249.3 ± 223.82	613.10 ± 539.00	30.29 ± 3.97	2.67 ± 0.15
Non-producer (*n* = 1)	Primary cultures	mMCB	2743.95 ± 36.98	155.29 ± 2.89	404.61 ± 3.02	17.67 ± 0.13	6.66 ± 0.23	5.90 ± 0.19	8.74 ± 0.09	4.87 ± 0.02
		mMCB_ISO_	2523.79 ± 25.44	167.85 ± 2.99	312.38 ± 4.2	15.04 ± 0.16	6.61 ± 0.07	5.61 ± 0.01	8.71 ± 0.26	4.71 ± 0.06
	Secondary cultures	mMCB_ISO_	3537.98 ± 33.75	1246.96 ± 14.86	687.74 ± 11.19	2.84 ± 0.02	73.89 ± 1.31	407.66 ± 10.76	7.16 ± 0.12	2.26 ± 0.05
	Control media	mMCB	1000.31 ± 5.09	36.90 ± 0.15	19.52 ± 0.80	27.11 ± 0.25	4.30 ± 0.10	0.23 ± 0.00	0.00 ± 0.00	2.29 ± 0.04
		mMCB_ISO_	1017.13 ± 22.67	36.63 ± 0.30	19.05 ± 0.39	27.77 ± 0.56	4.50 ± 0.10	0.32 ± 0.09	0.00 ± 0.00	2.41 ± 0.01

*mMCB* modified medium for colonic bacteria, *mMCB*_*ISO*_ modified medium for colonic bacteria supplemented with isoflavones

Key of statistical significance: mMCB compared to mMCB_ISO_: *p < 0.05

### Bacterial community analysis by PCR-DGGE

The structural diversity of the bacterial communities in the primary faecal cultures was initially investigated by PCR-DGGE. In samples from the mMCB_ISO_ medium inoculated with *S. equolifaciens* or *S. isoflavoniconvertens*, faint bands corresponding to the expected size for these species were appreciated (Additional file 1). Clear differences were seen in the bacterial DGGE patterns of the cultures for the different women. However, no changes were observed in the profiles for the mMCB and mMCB_ISO_ faecal cultures from the same woman. This suggested to us that the presence of isoflavones did not affect under the experimental culture conditions employed the dominant bacterial populations. Therefore, the bacterial communities of the secondary cultures were not further analysed using this technique.

### Bacterial community analysis by high-throughput DNA sequencing

**Fig. 2:**
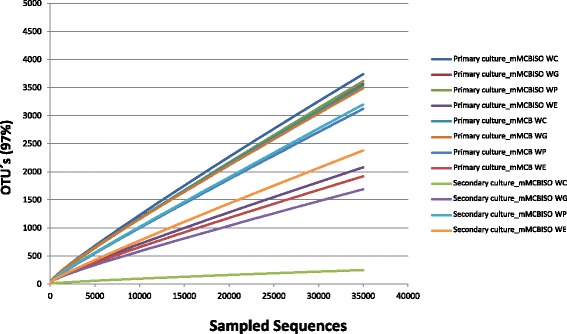
Rarefaction curves calculated for each sample of the study. Rarefaction curves at a 97% similarity level of partial sequences of the bacterial 16S rRNA gene from primary and secondary faecal cultures in media with (mMCB_ISO_) and without (mMCB) isoflavones

**Fig. 3:**
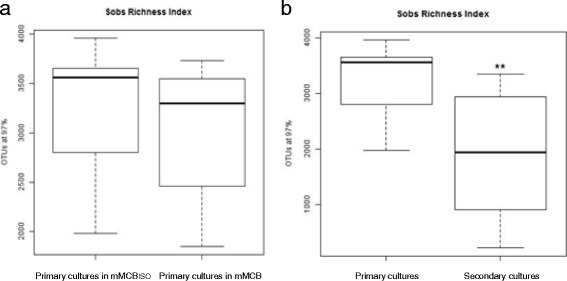
Differences in richness indices between faecal cultures. Comparison of Sobs, number of observed species, for the 16S rRNA gene sequences at 3% of divergence distance of: **a**) primary faecal cultures in media with (mMCB_ISO_) and without (mMCB) isoflavones, and **b**) primary and secondary cultures in media with isoflavones. *Asterisks* denoted statistically significant differences (Welch’s test, *p* value < 0.01)

The application of the Illumina technology allowed obtaining an average of 111,542 high quality PCR-amplicon sequences for each replicate. The number of high quality sequences among all the replicates ranged between 71,138–150,292, and estimated sample coverage was considered to be good for all libraries. The mean number of quality sequences retrieved per faecal culture, together with the numbers of OTUs, as defined by ≥97% sequence identity, and α-diversity indexes calculated for each sample are summarized in Additional file 2. To allow further comparative analyses between samples, each 16S rRNA gene amplicon library was rarefied to 35,000 sequences per replicate. The rarefaction curves for the normalized sequences showed that the secondary culture for subject WC (which did not render equol in vitro) was the least diverse (Fig. 2). Further, the numbers of OTUs obtained in all cultures (primary culture in mMCB, primary culture in mMCB_ISO_, and secondary culture in mMCB_ISO_) from the equol non-producer subject WE were smaller (2000–2300 OTUs) than for all other cultures (3000–3500 OTUs) (Additional file 2). Compared to the faecal cultures in mMCB, the presence of isoflavones had no significant influence on the richness index Sobs (number of observed species) (Fig. 3). However, a significant reduction of the bacterial diversity was recorded for the secondary cultures (*p* < 0.01).

**Fig. 4:**
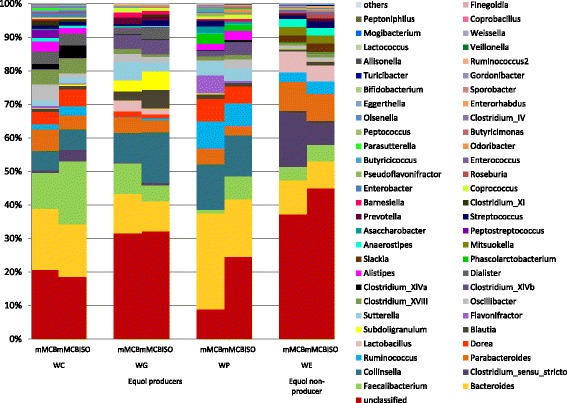
Bacterial composition at the genus level of primary faecal cultures. WC, WG and WP are equol producers meanwhile WE is an equol non-producer. Composition is represented as the mean relative abundance of the six replicates sequenced. Only genera contributing with a percentage > 0.05% of the total abundance in a least one sample are shown

Sequences were then taxonomically assigned using the RDP classifier. A majority of sequences in all samples belonged to the phylum Firmicutes (mean 55%), while sequences from Bacteroidetes (27%), Actinobacteria (9%) and Proteobacteria (7%) phyla were less abundant. At this taxonomic range, only 0.1% of the sequences remained unclassified/unassigned. At the genus-level, between 52 and 75 different genera were identified within each faecal culture. Among the dominant genera *Bacteroides* presented the highest percentage with a relative abundance between 10 and 20% of assigned reads; *Faecalibacterium* ranged between 5 and 10%; and sequences related to the *Clostridium “*sensu stricto*”* group also reached these percentages (5–10%), but only in the primary cultures from the equol non-producing woman (Fig. 4). Sequences assigned to the genus *Collinsella* also ranged within these abundances in the primary cultures from the equol producers.

Subdominant genera, varying in percentage from 1 to 5%, were represented by *Parabacteroides*, *Ruminococcus* and *Dorea*; the latter only in the primary cultures from equol producers. In agreement with the DGGE results, the bacterial composition profile obtained by 16S rDNA sequencing was quite similar for equivalent faecal cultures when grown with and without isoflavones (Fig. 4). Nonetheless, changes in subdominant, minor and assumingly rare genera were revealed by the high throughput DNA sequencing technique.

**Fig. 5:**
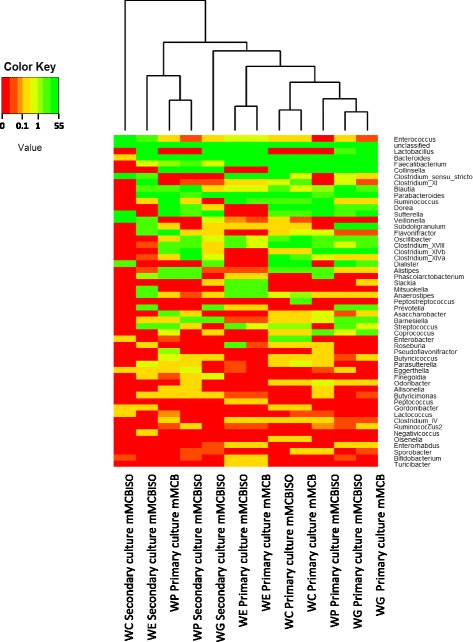
Heatmap based on mean relative abundances of major genera across primary and secondary faecal cultures. Legend: WC, WG and WP are equol producers meanwhile WE is an equol non-producer. Faecal cultures were grown in media with (mMCB_ISO_) and without (mMCB) isoflavones. Only genera contributing to >0.1% of the total abundance in at least one sample are represented in the vertical axis. Colour key: each colour represent a value range of relative abundance that was selected for optimal visualization ranging between 0 and 55%. Hierarchical clustering of samples was performed with the complete linkage and Euclidean distance through R package

A heatmap depicting relative abundances across samples (including primary and secondary cultures) is shown in Fig. 5. In general, primary faecal cultures in mMCB and mMCB_ISO_ from the same woman clustered together suggesting similar microbial profiles, as denoted previously by DGGE. On the other hand, the secondary cultures, with major changes in the bacterial communities’ patterns, plotted in a separate branche.

The application of the Metastats method (FDR correction 0.25) revealed that the presence of isoflavones in the culture medium altered the abundance at several taxonomic hierarchies. For example, the phylum Bacteroidetes was less represented in the presence of isoflavones (*p* = 0.001), in particular the family *Bacteroidaceae* with a decrease of reads from the genera *Bacteroides* and *Parabacteroides* (Additional file 3). On the contrary, the representation of the family *Ruminococcaceae* significantly increased with isoflavones (*p* = 0.011)*.* At the genus level, increases were recorded for *Roseburia* and *Odoribacter* sequences. Reads of these two genera were still significantly more abundant when excluding from analysis the faecal culture from the equol non-producing woman (WE). If omitting samples from this woman, and by comparing faecal primary cultures with secondary cultures in the presence of isoflavones, two genera belonging to the family *Coriobacteriaceae* (capable of synthesizing equol) were significantly increased, *Slackia* (*p* = 0.019) and *Eggerthella* (*p* = 0.030). Comparison of the microbial communities in the mMCB_ISO_ primary cultures for equol producers with that of the non-producer revealed a significant increase in the number of sequences belonging to 28 different genera (Additional file 4). The same number of genera showed an increased in relative abundance when comparing secondary cultures producing equol with those that did not (Additional file 5). Fifteen of the 28 genera (*Collinsella*, *Faecalibacterium*, *Dorea*, *Suterella*, *Clostridium* (groups XIVa and XIVb), *Alistipes*, *Oscillibacter*, *Barnesiella*, *Coprococcus*, *Finegoldia*, *Butyricicoccus*, *Asaccharobacter*, *Murdochiella*, *Allisonella* and *Odoribacter*) were found in both the primary and their derived secondary cultures, suggesting these groups to be more abundant in cultures yielding equol. Of note was the appearance of *Collinsella* (a member of the family *Coriobacteriaceae* not described as equol producer), which accounted for 6–15% of sequences in cultures that produced equol, while reads for this genus in isoflavone-containing cultures that did not yield equol was less than 0.04%. Furthermore, reads of the genus *Asaccharobacter,* a member of the same family harbouring equol-producing strains, increased significantly in all cultures producing equol.

**Fig. 6:**
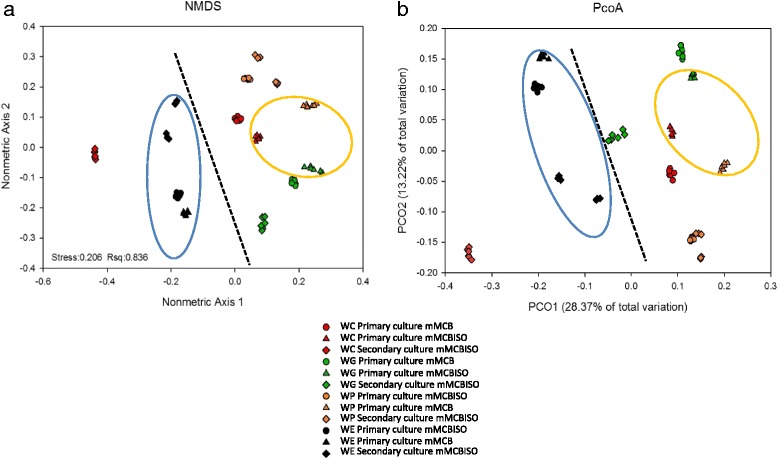
Clustering of operational taxonomic units (OTUs) of primary and secondary cultures. Legend: Non-metric multidimensional scaling (NMDS) (**a**), and principal coordinates analysis (PCoA) (**b**) showing clustering of the OTUs of the faecal cultures in this study. The *blue circle* embraces samples from the equol non-producing woman; the *yellow circle* includes primary cultures in medium with isoflavones from the equol-producing women; the *dotted line* separates samples that did not render equol

To visualize the overall differences between the bacterial community structures, multivariate statistical analyses, i.e., NMDS and PCoA were applied. This revealed that the bacterial communities of the women’s faecal primary and secondary cultures clustered together, with the exception of that of the secondary culture for woman WC (which, as stated above, showed a reduced diversity and did not produce equol) (Fig. 6). The cluster for the microbiota of this latter sample and those for the primary/secondary cultures for the equol non-producing subject (WE), lay at an appreciable distance from the cultures derived from equol producers (stress value 0.206 in NMDS). Further, the bacterial communities of the mMCB_ISO_ cultures for the equol- producing women showed more similarity to one another than to those grown in mMCB. AMOVA revealed the clustering of the two types of samples (in mMCB_ISO_ and mMCB) to be significantly different (*p* = 0.036).

Finally, OTUs significantly different in abundance between primary faecal cultures grown in in mMCB_ISO_ and in mMCB were manually assigned to the species level (sequence identity higher that 97%). In cultures grown with isoflavones, OTUs belonging to *Faecalibacterium prausnitzii, Eubacterium halli, Subdoligranulum variabile, Ruminococcus flavefaciens/callidus, Blautia obeum* and *Bacteroides xylanisolvens/ovatus* were increased (Additional file 6).

## Discussion

In this work, the bacterial diversity and metabolic changes in cultures of faecal samples from isoflavone-treated menopausal women were investigated. For that purpose, an in vitro anaerobic batch culture system in a modified medium for colonic bacteria supplemented or not with isoflavones was developed. Production of equol was determined with a recently described UHPLC method [17] and verified by inoculating the isoflavone-containing medium with *S. equolifaciens*, an equol-producing bacterium*.* Similar levels of equol production to those obtained with this control strain were recorded in the primary faecal cultures for women with an equol producer phenotype (as determined previously by equol urinary excretion [14]) - but not in the faecal cultures from a non-equol producer. This strongly indicates differences among the faecal samples in types and/or numbers of intestinal bacteria with capacity to produce equol. The stability of isoflavone bioconversion into equol was further tested by making subcultures of the faecal primary cultures in fresh media. In the presence of isoflavones, equol production was maintained in all except one of these secondary cultures, suggesting that, under the experimental conditions, bacterial types capable of metabolizing isoflavones and producing equol maintain viability and the equol-producing capability, which opens possibilities for a rational propagation and selection of these bacteria. It should be noted that with the UHPLC methodology used in this study other daidzein-derived metabolites that could be formed in the faecal cultures, such as hydroxydadidzein (DHD) and *O*-desmethylangolensin (*O*-DMA), might have passed undetected.

In our study, the bacterial composition of the faecal primary and secondary cultures was characterized by two culture-independent methods. PCR-DGGE analysis revealed no major changes in the bacterial communities between the primary cultures with and without isoflavones. This would suggest that the dominant gut populations are a priori neither involved in the metabolism of isoflavones nor influenced by the presence of these compounds. However, the 16S rDNA sequencing data revealed significant changes in different taxa in the presence of isoflavones, particularly with respect to minority groups, which cannot be tracked by the DGGE technique. Although the diversity observed in the faecal cultures was similar in the media with and without isoflavones, a larger number of OTUs was scored in samples yielding equol. This suggests a more diverse bacterial community coming from the faeces of women with an equol producer status.

*Collinsella* was one of the genera that increased in abundance considerably, both in the primary and secondary cultures that produced equol. This genus belongs to *Coriobacteriaceae*, a family that harbours most of the currently described equol-producing bacteria [7]. Increases in the number of *Collinsella* sequences in response to isoflavone treatment have recently been reported in vivo [28]. Although to a lesser extent, the number of reads associated with the genus *Asaccharobacter* also increased significantly in the faecal cultures producing equol. This agrees with the fact that the single species of this genus, *Asaccharobacter celatus*, has been reported to be an equol producer [29]. Other intestinal bacteria recently associated with isoflavone metabolism in humans, such as *Dorea* and *Finegoldia* [28, 30], were also significantly increased in all cultures producing equol. In agreement with the present results, isoflavones have further been described to stimulate in vivo majority microorganisms of the *Clostridium* clusters XIVa and IV, to the latter of which *F. prausnitzii* belongs [31, 14]. On the other hand, like other polyphenols, isoflavones may have antimicrobial activity, which could modulate the diversity and composition of the faecal bacterial communities [10]. In this work, addition of isoflavones to the faecal cultures drove to a decrease of some taxa. In particular, the genera *Bacteroides* and *Parabacteroides* reduced their numbers in the presence of these compounds.

Papers reporting production of equol in slurry cultures inoculated with faeces from equol producing subjects have been published previously [12, 13, 30]. However, as far as we are aware, this is the first study that making use of a soy isoflavone extract (commercial formulation) added directly to a colonic culturing medium, describes in deep the bacterial changes and response to isoflavone enrichment by high-throughput 16S DNA sequencing. Although this, the presence in the supplement of other minor components apart from isoflavones (excipients) that might influence the faecal bacterial communities cannot be completely ruled out.

Production of SCFAs (relevant gut bacterial metabolites) was determined in the batch faecal cultures in relation with the addition of isoflavones and the production of equol. SCFAs production in the colon results from bacterial fermentation of dietary starches, fibre and sugars [32]. The SCFAs acetic (C2), propionic (C3) and butyric (C4) acids are the main fermentation end-products in the gut, and are thought to have anti-inflammatory and anti-carcinogenic activities [33]. In contrast, BCFAs, such as isobutyric and isovaleric acids, often associated with protein breakdown, have been less studied. The present data reveal changes in SCFA production in the presence of isoflavones. The increased quantities of the main SCFAs (acetic, propionic and butyric) and BCFAs in the faecal cultures with isoflavones indicates microbial activity leading to the production of these compounds. The soy isoflavone-glycosides can be initially deconjugated by endogenous intestinal bacteria with hydrolytic activity (probably β-glucosidases), as it was observed in this study with the reduction in the daidzin levels after cultivation. This activity releases isoflavone aglycones [34], but also sugars that may provide an alternative energy source to the cultures. This would agree with some studies involving prebiotic-related compounds such as beta-glucan or cellobiose, which have been shown to increase BCFAs production [35, 36]. At the genus level, *Roseburia* and *Odoribacter* sequences were both significantly increased in mMCB_ISO_ as compared to the medium without isoflavones. Reduction in members of these two genera has been suggested to enhance inflammation by decreasing SCFA availability [37, 38]. Although not statistically significant, butyrate production in this work was greater in the faecal cultures that produced equol. Butyrate-producing bacteria such as *Roseburia*, *E. hallii* (both from Clostridium cluster XIVa) and *F. prausnitzii* were more abundant when faecal cultures were incubated with isoflavones, particularly in those that produce equol. When significant OTUs were assigned manually at the species level other cellulolytic butyrate producers, such as *R. flavefaciens* and *S. variabile* (both of the *Clostridium* cluster IV) [37], and propionate producers from the *Clostridium* cluster XIVa, such as *B. obeum*, were also identified.

Cross-feeding mechanisms among the more diverse faecal microbiota in equol producers might further be partly responsible for the increased production of propionic and butyric acids in isoflavone-containing cultures. Acetate is the predominant SCFA produced by bacterial fermentation in the gut, and both, *F. prausnitzii* and *E. hallii* (the sequences of which became more abundant when cultures were grown in the presence of isoflavones) are well known acetate-utilizing, butyrate-producing species [33].

Finally, isoflavone metabolism might also be affected by the presence of SCFAs; i.e., butyric acid has been shown to enhance equol production by *A. celatus* [29], and an increase production of equol by a mixed microbial culture from human faeces in the presence of propionate and butyrate has also been reported [30].

## Conclusions

Faecal cultures from women with an equol producer phenotype yielded equol in vitro in faecal fermentations with isoflavones, suggesting the presence in faeces of active bacterial types able to produce this compound. The application of high-throughput DNA sequencing of 16S rRNA gene PCR amplicons revealed the composition of the faecal bacterial communities to be modified by the presence of isoflavones, including increases in equol-producing taxa. Although limited by the low number of cultures, isoflavones seem also to promote, at least in vitro, SCFAs production. This increase might be due to the growth stimulation of specific SCFA-producing bacterial types from the *Ruminococaceae* (members of the *Clostridium* cluster IV) and *Lachnospiraceae* (*Clostridium* cluster XIVa) families.

## Additional files

Additional file 1: Effect of isoflavones on dominant bacterial populations determined by DGGE. PCR-DGGE profiles of the primary faecal cultures from equol-producing women grown in modified medium for colonic bacteria supplemented (mMCB_ISO_) or not (mMCB) with isoflavones; A) WC samples, B) WG samples. M: DGGE marker [comprising the species *Slackia isoflavoniconvertens* (1) and *Slackia equolifaciens* (2)]. (PPTX 164 kb)

Additional file 2: Summary of sequence processing. Number of quality reads, sample coverage, richness estimators and diversity indexes of 16S rDNA libraries of primary and secondary faecal cultures. (DOCX 20 kb)

Additional file 3: Effect of isoflavones in microbial abundance. Families and genera showing significant (*p* value <0.05) increases (grey) and decreases in their relative abundances (% sequences) when comparing primary faecal cultures in medium with and without isoflavones. (DOCX 16 kb)

Additional file 4: Differences in microbial genera associated with equol production in primary faecal cultures. Genera showing significant increases (*p* value <0.05) in their relative abundances (% sequences) in medium with isoflavones when comparing primary cultures from non-producer and equol producer women. (DOCX 16 kb)

Additional file 5: Differences in microbial genera associated with equol production in secondary faecal cultures. Genera showing significant increases (*p* value <0.05) in their relative abundances (% sequences) in medium with isoflavones when comparing secondary cultures that rendered equol production with those that did not. (DOCX 17 kb)

Additional file 6: Effect of isoflavones in microbial species abundance. Identification of OTUs (3% distance level) showing significant increases (*p* value <0.05) in their relative abundances (%) in primary faecal cultures in mMCB_ISO_ as compared to that in mMCB. (DOCX 17 kb)

## Abbreviations

AMOVA: Analysis of molecular variance
ANOSIM: Analysis of similarities
BCFAs: Branched chain fatty acids
DGGE: Denaturing gradient gel electrophoresis
EFSA: European Food Safety Authority
FDR: False discovery rate
GC: Gas chromatography
mMCB: modified medium for colonic bacteria
mMCB_ISO_: modified medium for colonic bacteria supplemented with isoflavones
NMDS: Non-metric multidimensional scaling
OTUs: Operational taxonomic units
PBS: Phosphate buffered saline
PCoA: Principal coordinates analysis
PDA: Photodiode array
RDP: Ribosomal database project
SCFAs: Short-chain fatty acids
SOP: Standard operating procedure
UHPLC: Ultra-high-performance liquid chromatography
