# Supplementary figures and images for: Bacterial communities and metabolic activity of faecal cultures from equol producer and non-producer menopausal women under treatment with soy isoflavones
Source: BMC Microbiol. 2017 Apr 17;17:93. doi: 10.1186/s12866-017-1001-y (PMC5392999; doi:10.1186/s12866-017-1001-y)

## Slide 1
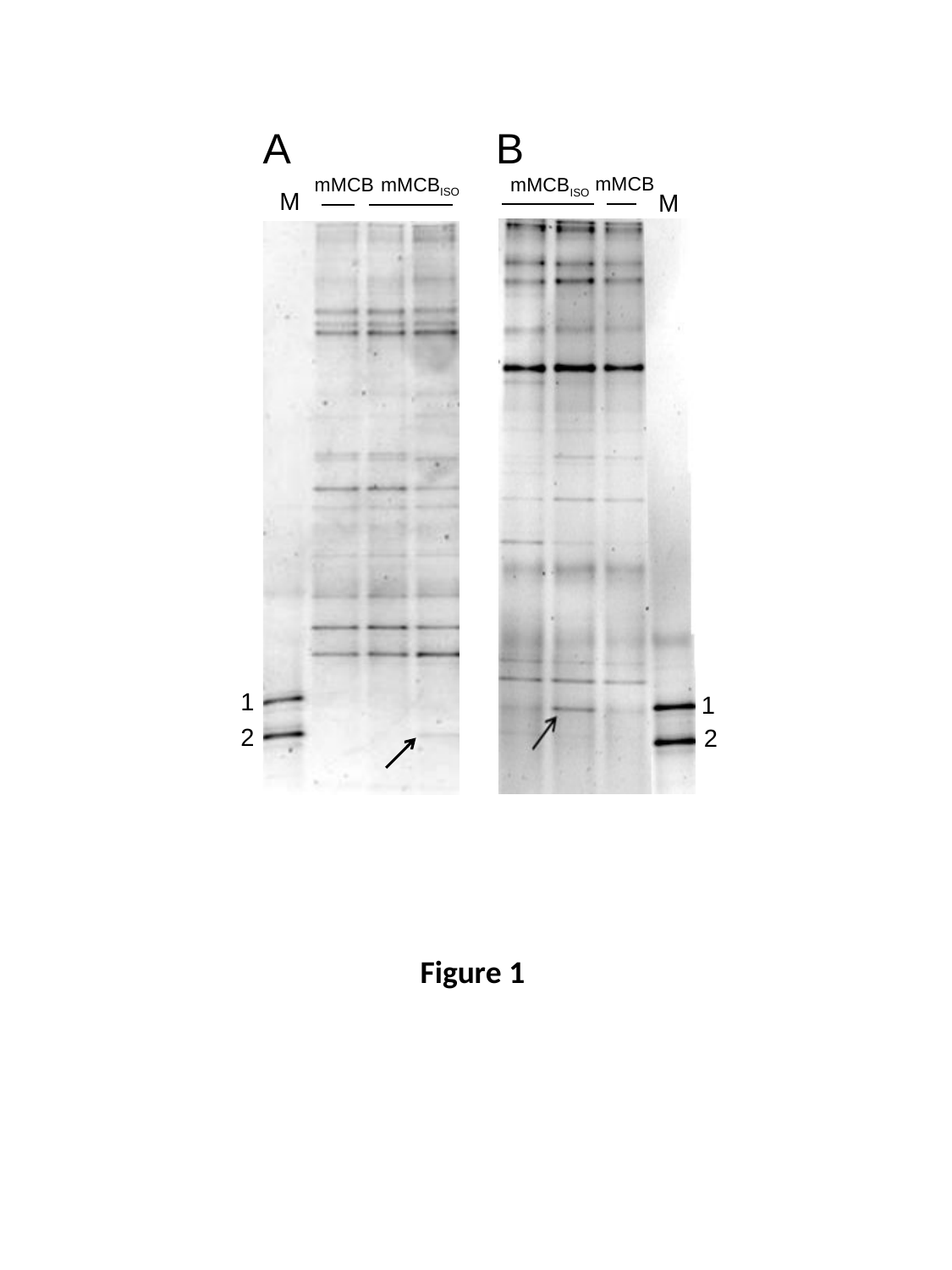

A
B
mMCB
mMCB
mMCBISO
mMCBISO
M
M
1
1
2
2
Figure 1

Supplement: Supplementary file 1 — Effect of isoflavones on dominant bacterial populations determined by DGGE. PCR-DGGE profiles of the primary faecal cultures from equol-producing women grown in modified medium for colonic bacteria supplemented (mMCBISO) or not (mMCB) with isoflavones; A) WC samples, B) WG samples. M: DGGE marker [comprising the species Slackia isoflavoniconvertens (1) and Slackia equolifaciens (2)]. (PPTX 164 kb) [file 12866_2017_1001_MOESM1_ESM.pptx]
