# Supplementary material for: Bacterial communities and metabolic activity of faecal cultures from equol producer and non-producer menopausal women under treatment with soy isoflavones
Source: BMC Microbiol. 2017 Apr 17;17:93. doi: 10.1186/s12866-017-1001-y (PMC5392999; doi:10.1186/s12866-017-1001-y)
Supplement: Supplementary file 2 — Summary of sequence processing. Number of quality reads, sample coverage, richness estimators and diversity indexes of 16S rDNA libraries of primary and secondary faecal cultures. (DOCX 20 kb) [file 12866_2017_1001_MOESM2_ESM.docx]

**Summary of sequence processing.** Number of quality reads, sample coverage, richness estimators and diversity indexes of 16S rDNA libraries of the primary and secondary faecal cultures.

| Library | | NS | OTUs^a^ | Chao1^b^ | Simpson^b^ | Shannon^b^ | ESC^c^ |
| --- | --- | --- | --- | --- | --- | --- | --- |
| WC | Primary culture mMCB | 113,343 | 3,538 | 42,912.87 (36,262.24; 50,915.37) | 0.03 (0.03; 0.03) | 4.66 (4.64; 4.68) | 0.91 |
|  | Primary culture mMCB_ISO_ | 117,798 | 3,738 | 39,743.64 (34,111.65; 46,421.64) | 0.03 (0.03; 0.03) | 4.54 (4.51; 4.56) | 0.90 |
|  | Secondary culture mMCB_ISO_ | 102,101 | 249 | 1,120.57 (774.96; 1,694.67) | 0.36 (0.35; 0.36) | 1.47 (1.46; 1.49) | 0.99 |
| WG | Primary culture mMCB | 122,186 | 3,489 | 45,965.86 (38,489.01; 55,041.67) | 0.03 (0.03; 0.03) | 4.47 (4.45; 4.5) | 0.91 |
|  | Primary culture mMCB_ISO_ | 93,477 | 3,562 | 49,825.82 (41,588.49; 59,851.52) | 0.05 (0.05; 0.05) | 4.32 (4.29; 4.35) | 0.91 |
|  | Secondary culture mMCB_ISO_ | 105,583 | 1,686 | 18,315.58 (14,525.55; 23,227.37) | 0.15 (0.15; 0.15) | 3.12 (3.09; 3.14) | 0.96 |
| WP | Primary culture mMCB | 113,924 | 3,120 | 47,705.44 (39,083.6; 58,400.07) | 0.05 (0.05; 0.05) | 4.06 (4.04; 4.09) | 0.92 |
|  | Primary culture mMCB_ISO_ | 114,540 | 3,612 | 65,179.68 (53,228.06; 80,017.35) | 0.05 (0.05; 0.05) | 4.26 (4.24; 4.29) | 0.90 |
|  | Secondary culture mMCB_ISO_ | 101,670 | 3,197 | 62,336.79 (49,903.97; 78,089.56) | 0.05 (0.05; 0.05) | 4.11 (4.09; 4.14) | 0.91 |
| WE | Primary culture mMCB | 115,916 | 1,918 | 18,791.06 (15,277.95; 23,230.43) | 0.08 (0.07; 0.08) | 3.46 (3.44; 3.49) | 0.95 |
|  | Primary culture mMCB_ISO_ | 127,122 | 2,077 | 23,154.77 (18,701.53; 28,804.76) | 0.07 (0.07; 0.07) | 3.66 (3.63; 3.68) | 0.95 |
|  | Secondary culture mMCB_ISO_ | 110,850 | 2,379 | 25,867.57 (21,411.11; 31,371.44) | 0.05 (0.05; 0.05) | 3.8 (3.78; 3.82) | 0.94 |

Abbreviations: ESC, estimated sample coverage; NS, average number of quality sequences for each sample; OTU, operational taxonomic unit

WC, WG and WP are equol-producing women; WP is a equol non-producing woman

^a^ Calculated by MOTHUR at the 3% distance level

^b^ Diversity indexes calculated using MOTHUR (3% distance)

Values in brackets are 95% confidence intervals as calculated by MOTHUR

^c^ ESC: Cx= 1 – (Nx/n), where Nx is the number of unique sequence and n is the total number of sequences

All calculations (a, b and c) were made with a subsample of 35,000 sequences per library
