# Supplementary material for: Bacterial communities and metabolic activity of faecal cultures from equol producer and non-producer menopausal women under treatment with soy isoflavones
Source: BMC Microbiol. 2017 Apr 17;17:93. doi: 10.1186/s12866-017-1001-y (PMC5392999; doi:10.1186/s12866-017-1001-y)
Supplement: Supplementary file 3 — Effect of isoflavones in microbial abundance. Families and genera showing significant (p value <0.05) increases (grey) and decreases in their relative abundances (% sequences) when comparing primary faecal cultures in medium with and without isoflavones. (DOCX 16 kb) [file 12866_2017_1001_MOESM3_ESM.docx]

**Effect of isoflavones in microbial abundance.** Families and genera showing significant (*p* value <0.05) increases (grey) and decreases in their relative abundances (% sequences) when comparing primary faecal cultures in medium with and without isoflavones.

|  |  | Primary cultures  mMCB^a^ | Primary cultures  mMCB_ISO_^a^ |
| --- | --- | --- | --- |
| Family | *p*-value^b^ | %  relative abundance^c^ | %  relative abundance^c^ |
| *Ruminococcaceae* | 0.011 | 24.214±1.484 | 30.167±1.667 |
| *Bacteroidaceae* | 0.010 | 17.144±1.512 | 12.452±0.847 |
| *Porphyromonadaceae* | 0.007 | 6.853±0.268 | 5.656±0.350 |
| *Clostridiales_Incertae_Sedis*_XI | 0.015 | 0.038±0.011 | 0.009±0.002 |
| Genus |  |  |  |
| *Roseburia* | 0.001 | 0.060±0.011 | 0.470±0.083 |
| *Odoribacter* | 0.002 | 0.088±0.013 | 0.257±0.054 |
| *Bacteroides* | 0.016 | 17.144±1.512 | 12.452±0.847 |
| *Parabacteroides* | 0.018 | 6.088±0.341 | 4.721±0.433 |
| *Flavonifractor* | 0.019 | 1.543±0.468 | 0.281±0.055 |
| *Peptostreptococcus* | 0.020 | 0.587±0.213 | 0.002±0.001 |
| *Butyricicoccus* | 0.005 | 0.410±0.067 | 0.174±0.037 |
| *Pseudoflavonifractor* | 0.025 | 0.262±0.092 | 0.052±0.018 |
| *Finegoldia* | 0.011 | 0.019±0.005 | 0.006±0.002 |
| *Peptoniphilus* | 0.012 | 0.017±0.006 | 0.001±0.001 |

^a^mMCB: modified medium for colonic bacteria; mMCBISO: modified medium for colonic bacteria supplemented with isoflavones

^b^Significance was considered below a *p*-value of 0.05, multiple hypothesis tests correction of Benjamini and Hochberg was applied with a FDR=0.25.

^c^Mean relative abundance ± standard deviation.
