# Supplementary material for: Bacterial communities and metabolic activity of faecal cultures from equol producer and non-producer menopausal women under treatment with soy isoflavones
Source: BMC Microbiol. 2017 Apr 17;17:93. doi: 10.1186/s12866-017-1001-y (PMC5392999; doi:10.1186/s12866-017-1001-y)
Supplement: Supplementary file 4 — Differences in microbial genera associated with equol production in primary faecal cultures. Genera showing significant increases (p value <0.05) in their relative abundances (% sequences) in medium with isoflavones when comparing primary cultures from non-producer and equol producer women. (DOCX 16 kb) [file 12866_2017_1001_MOESM4_ESM.docx]

**Differences in microbial genera associated with equol production in primary faecal cultures.** Genera showing significant increases (*p* value <0.05) in their relative abundances (% sequences) in medium with isoflavones when comparing primary cultures from non-producer and equol producer women.

|  |  | Primary culture equol non-producer woman | Primary cultures equol producers |
| --- | --- | --- | --- |
| Genus | *p*-value^a^ | %  relative abundance^b^ | %  relative abundance |
| *Collinsella* | 0.000 | 0.004±0.002 | 11.098±0.970 |
| *Bacteroides* | 0.000 | 8.142±0.180 | 13.888±0.898 |
| *Faecalibacterium* | 0.007 | 4.781±0.002 | 10.160±1.523 |
| *Dorea* | 0.000 | 0.013±0.002 | 3.808±0.446 |
| *Clostridium* group XlVb | 0.000 | 0.006±0.002 | 2.745±0.458 |
| *Sutterella* | 0.000 | 0.003±0.001 | 2.682±0.207 |
| *Dialister* | 0.000 | 0.002±0.001 | 2.279±0.392 |
| *Blautia* | 0.011 | 0.894±0.035 | 2.564±0.511 |
| *Alistipes* | 0.000 | 0.066±0.009 | 1.549±0.243 |
| *Clostridium* group XlVa | 0.004 | 0.019±0.006 | 1.483±0.384 |
| *Coprococcus* | 0.000 | 0.007±0.002 | 0.828±0.107 |
| *Oscillibacter* | 0.004 | 1.151±0.104 | 1.867±0.153 |
| *Barnesiella* | 0.002 | 0.092±0.009 | 0.641±0.112 |
| *Enterobacter* | 0.027 | 0.002±0.001 | 0.449±0.154 |
| *Asaccharobacter* | 0.005 | 0.020±0.005 | 0.368±0.094 |
| *Flavonifractor* | 0.001 | 0.033±0.005 | 0.364±0.061 |
| *Odoribacter* | 0.002 | 0.041±0.005 | 0.329±0.064 |
| *Butyricicoccus* | 0.003 | 0.030±0.005 | 0.223±0.044 |
| *Parasutterella* | 0.003 | 0.051±0.012 | 0.240±0.042 |
| *Olsenella* | 0.044 | 0.001±0.001 | 0.104±0.037 |
| *Gordonibacter* | 0.008 | 0.004±0.002 | 0.066±0.018 |
| *Lactococcus* | 0.002 | 0.002±0.001 | 0.041±0.008 |
| *Allisonella* | 0.034 | 0.000±0.000 | 0.038±0.014 |
| *Weissella* | 0.046 | 0.001±0.001 | 0.032±0.011 |
| *Coprobacillus* | 0.048 | 0.000±0.000 | 0.008±0.003 |
| *Finegoldia* | 0.021 | 0.001±0.001 | 0.008±0.002 |
| *Murdochiella* | 0.011 | 0.000±0.000 | 0.003±0.001 |
| *Atopobium* | 0.028 | 0.000±0.000 | 0.002±0.001 |

^a^Significance was considered below a *p*-value of 0.05, multiple hypothesis tests correction of Benjamini and Hochberg was applied with a FDR=0.25.

^b^Mean relative abundance ± standard deviation.
