# Supplementary material for: Bacterial communities and metabolic activity of faecal cultures from equol producer and non-producer menopausal women under treatment with soy isoflavones
Source: BMC Microbiol. 2017 Apr 17;17:93. doi: 10.1186/s12866-017-1001-y (PMC5392999; doi:10.1186/s12866-017-1001-y)
Supplement: Supplementary file 5 — Differences in microbial genera associated with equol production in secondary faecal cultures. Genera showing significant increases (p value <0.05) in their relative abundances (% sequences) in medium with isoflavones when comparing secondary cultures that rendered equol production with those that did not. (DOCX 17 kb) [file 12866_2017_1001_MOESM5_ESM.docx]

**Differences in microbial genera associated with equol production in secondary faecal cultures.** Genera showing significant increases (*p* value <0.05) in their relative abundances (% sequences) in medium with isoflavones when comparing secondary cultures that rendered equol production with those that did not.

|  |  | Secondary cultures equol^-^ | Secondary cultures equol^+^ |
| --- | --- | --- | --- |
| Genus | *p*-value^a^ | %  relative abundance^b^ | %  relative abundance |
| *Lactobacillus* | 0.044 | 4.802±1.528 | 17.616±5.420 |
| *Collinsella* | 0.001 | 0.025±0.006 | 12.406±1.819 |
| *Sutterella* | 0.001 | 1.669±0.507 | 4.665±0.613 |
| *Clostridium* group XlVb | 0.001 | 0.005±0.001 | 2.082±0.586 |
| *Clostridium* group XlVa | 0.001 | 0.040±0.012 | 1.878±0.471 |
| *Oscillibacter* | 0.001 | 0.022±0.009 | 1.836±0.375 |
| *Barnesiella* | 0.001 | 0.060±0.019 | 1.586±0.187 |
| *Alistipes* | 0.001 | 0.047±0.013 | 1.474±0.444 |
| *Dorea* | 0.001 | 0.003±0.001 | 1.229±0.354 |
| *Clostridium* group XVIII | 0.001 | 0.031±0.009 | 0.981±0.264 |
| *Faecalibacterium* | 0.013 | 0.473±0.147 | 1.346±0.301 |
| *Finegoldia* | 0.001 | 0.069±0.031 | 0.487±0.086 |
| *Butyricicoccus* | 0.001 | 0.023±0.009 | 0.313±0.051 |
| *Allisonella* | 0.007 | 0.000±0.000 | 0.266±0.095 |
| *Odoribacter* | 0.009 | 0.012±0.004 | 0.261±0.094 |
| *Ruminococcus2* | 0.001 | 0.001±0.000 | 0.194±0.059 |
| *Coprococcus* | 0.001 | 0.001±0.000 | 0.177±0.053 |
| *Slackia* | 0.022 | 0.020±0.006 | 0.193±0.059 |
| *Asaccharobacter* | 0.001 | 0.006±0.002 | 0.149±0.048 |
| *Sporobacter* | 0.001 | 0.001±0.000 | 0.071±0.009 |
| *Pseudoflavonifractor* | 0.013 | 0.000±0.000 | 0.014±0.006 |
| *Murdochiella* | 0.001 | 0.000±0.000 | 0.010±0.004 |
| *Anaerotruncus* | 0.001 | 0.000±0.000 | 0.007±0.001 |
| *Mogibacterium* | 0.048 | 0.000±0.000 | 0.003±0.001 |
| *Anaerovorax* | 0.015 | 0.000±0.000 | 0.003±0.002 |
| *Sphingopyxis* | 0.001 | 0.000±0.000 | 0.003±0.001 |
| *Actinomyces* | 0.039 | 0.000±0.000 | 0.002±0.001 |
| *Eubacterium* | 0.031 | 0.000±0.000 | 0.001±0.001 |

^a^Significance was considered below a *p*-value of 0.05, multiple hypothesis tests correction of Benjamini and Hochberg was applied with a FDR=0.25.

^b^Mean relative abundance ± standard deviation.
