# Supplementary material for: Bacterial communities and metabolic activity of faecal cultures from equol producer and non-producer menopausal women under treatment with soy isoflavones
Source: BMC Microbiol. 2017 Apr 17;17:93. doi: 10.1186/s12866-017-1001-y (PMC5392999; doi:10.1186/s12866-017-1001-y)
Supplement: Supplementary file 6 — Effect of isoflavones in microbial species abundance. Identification of OTUs (3% distance level) showing significant increases (p value <0.05) in their relative abundances (%) in primary faecal cultures in mMCBISO as compared to that in mMCB. (DOCX 17 kb) [file 12866_2017_1001_MOESM6_ESM.docx]

**Effect of isoflavones in microbial species abundance.** Identification of OTUs (3% distance level) showing significant increases (*p* value <0.05) in their relative abundances (%) in primary faecal cultures in mMCB_ISO_ as compared with mMCB.

| OTU Code | Primary cultures  mMCB | | Primary cultures  mMCB_ISO_ | | *p*-value | ^a^Blast match  (closest relative) | Similarity  (%) | Family | Cluster |
| --- | --- | --- | --- | --- | --- | --- | --- | --- | --- |
|  | mean | standard deviation | mean | standard deviation |  |  |  |  |  |
| Otu099029 | 0.00024 | 0.00016 | 0.00274 | 0.00087 | 0.00002 | *Faecalibacterium prausnitzii* | 98 | *Ruminococaceae* | cluster IV |
| Otu131074 | 0.00000 | 0.00000 | 0.00214 | 0.00122 | 0.00001 | *Ruminococcus flavefaciens/callidus* | 97 | *Ruminococaceae* | cluster IV |
| Otu124213 | 0.00000 | 0.00000 | 0.00226 | 0.00126 | 0.00000 | *Subdoligranulum variabile* | 98 | *Ruminococaceae* | cluster IV |
| Otu022359 | 0.00012 | 0.00012 | 0.00250 | 0.00101 | 0.00001 | *Eubacterium hallii* | 99 | *Lachnospiraceae* | cluster XIVa |
| Otu067918 | 0.00000 | 0.00000 | 0.00226 | 0.00226 | 0.00000 | *Blautia obeum* | 98 | *Lachnospiraceae* | cluster XIVa |
| Otu148782 | 0.00000 | 0.00000 | 0.00238 | 0.00238 | 0.00000 | *Bacteroides xylanisolvens/ovatus* | 99 | *Bacteroidaceae* |  |

Abbreviations: OTU, operational taxonomic unit; mMCB: modified medium for colonic bacteria; mMCBISO, modified medium for colonic bacteria supplemented with isoflavones.

^a^OTUs were assigned at species level through sequence comparison against the Greengenes 16S rRNA gene database only when showing a nucleotide identity 97%.
